# Severe Acrodermatitis Continua of Hallopeau With Atypical Dermoscopic Features: A Diagnostic Pitfall

**DOI:** 10.7759/cureus.116047

**Published:** 2026-09-10

**Authors:** Aimane Zaim, Hanane Baybay, Samya Berrada, Layla Tahiri Elousrouti, Fatima Zahra Mernissi

**Affiliations:** 1 Department of Dermatology, Hassan II University Hospital, Sidi Mohamed Ben Abdellah University, Fez, MAR; 2 Department of Pathology, Hassan II University Hospital, Sidi Mohamed Ben Abdellah University, Fez, MAR

**Keywords:** acrodermatitis continua of hallopeau, anonychia, chronic paronychia, dermoscopy, nail apparatus, nail erosive lichen planus, pustular psoriasis

## Abstract

Acrodermatitis continua of Hallopeau is a rare, chronic, relapsing form of localized pustular psoriasis characterized by painful sterile pustules and progressive destruction of the nail apparatus. Its clinical and dermoscopic features may be atypical, leading to diagnostic delays and inappropriate treatment. We present the case of a 78-year-old man with a one-year history of painful hyperkeratotic lesions affecting several fingers, with recent worsening, extension, and purulent discharge. Examination revealed bilateral chronic paronychia with peripheral erosions involving both thumbs and the left index finger, covered by thick yellow-green hyperkeratosis and purulent discharge. Proximal erythematous, crusted plaques extended to the digit bases. Chronic fungal paronychia with secondary bacterial infection was initially suspected. Bacterial and fungal cultures from purulent discharge and erosive areas were negative, although* Pseudomonas aeruginosa* was isolated from the extracted hyperkeratotic material. Following local care and antimicrobial therapy, the hyperkeratosis cleared, revealing anonychia and a markedly inflamed, erosive periungual surface. Dermoscopy showed large structureless whitish areas corresponding to giant pustules, coalescent milky pustules, yellowish crusts, whitish scales, and regularly distributed dotted, globular, and glomerular vessels. Shiny reticular white structures and small polymorphous white-yellow structures were also observed, mimicking nail erosive lichen planus and candidiasis. Histopathology demonstrated psoriasiform acanthosis, laminated parakeratosis, Munro microabscesses, dilated papillary dermal capillaries, and a neutrophilic and lymphohistiocytic infiltrate without interface changes, confirming acrodermatitis continua of Hallopeau with secondary bacterial superinfection. Acrodermatitis continua of Hallopeau should be considered in the differential diagnosis of chronic, destructive paronychia, particularly when accompanied by nail dystrophy or anonychia. Massive hyperkeratosis and atypical dermoscopic findings may obscure the diagnosis and mimic other inflammatory or infectious disorders. Accurate diagnosis requires integration of clinical, dermoscopic, microbiologic, and histopathologic findings to enable timely and appropriate management.

## Introduction

Acrodermatitis continua of Hallopeau (ACH) is a rare, chronic, and relapsing inflammatory pustular dermatosis primarily involving the nail apparatus. Historically, it has been described under various terms, including acrodermatitis continua suppurativa, pustular acrodermatitis, acropustulosis, acrodermatitis perstans, dermatitis perstans, and dermatitis repens. According to the European Rare and Severe Psoriasis Expert Network (ERASPEN), ACH represents a distinct subtype within the spectrum of pustular psoriasis (PP), alongside generalized pustular psoriasis (GPP) and palmoplantar pustulosis (PPP), with nail apparatus involvement considered a defining feature [1].

Clinically, ACH presents with persistent, painful paronychia. Typically, symptoms begin with the tip of one digit turning erythematous and subsequently developing tender sterile pustules that migrate under the nail bed and matrix. Disease progression may lead to onychodystrophy, anonychia, and, in long-standing severe cases, distal phalanx osteolysis. The disease usually remains confined to the digit(s) for months or years before progressing to affect other digits or extend proximally to the dorsal hand or foot. Evolution into GPP has also been reported [1-3]. In these ways, ACH can induce severe impairment and have a significant negative influence on patients' quality of life; hence, an early and prompt diagnosis is required.

At the molecular level, ACH has been associated with mutations in IL-36RN (~20% of cases), as well as CARD14 and AP1S3, resulting in dysregulated IL-36 signaling, neutrophilic epidermal infiltration, and pustule formation [1]. Despite advances in understanding its pathophysiology, ACH remains diagnostically challenging and therapeutically refractory. Atypical clinical presentations can further obscure recognition, delaying appropriate management.

We report a case of ACH with an unusual presentation and misleading dermoscopic features, highlighting diagnostic pitfalls of this uncommon condition.

## Case presentation

A 78-year-old man with a history of heart failure, hypertension, and diabetes mellitus presented with a one-year history of painful hyperkeratotic lesions affecting several fingers. Two months before presentation, the lesions had worsened, with increased pain, progressive extension, and purulent discharge. He denied systemic symptoms or functional impairment.

Dermatologic examination revealed bilateral chronic paronychia with peripheral erosions involving both thumbs and the left index finger. The lesions were covered by thick yellow-green hyperkeratosis with purulent discharge upon pressure. Proximally, erythematous plaques with irregular crusted surfaces extended to the digit bases (Figure 1). Nail examination showed pachyxanthonychia of the toenails and onychogryphosis of the right index. No other cutaneous, scalp, or mucosal lesions were observed.

**Figure 1:**
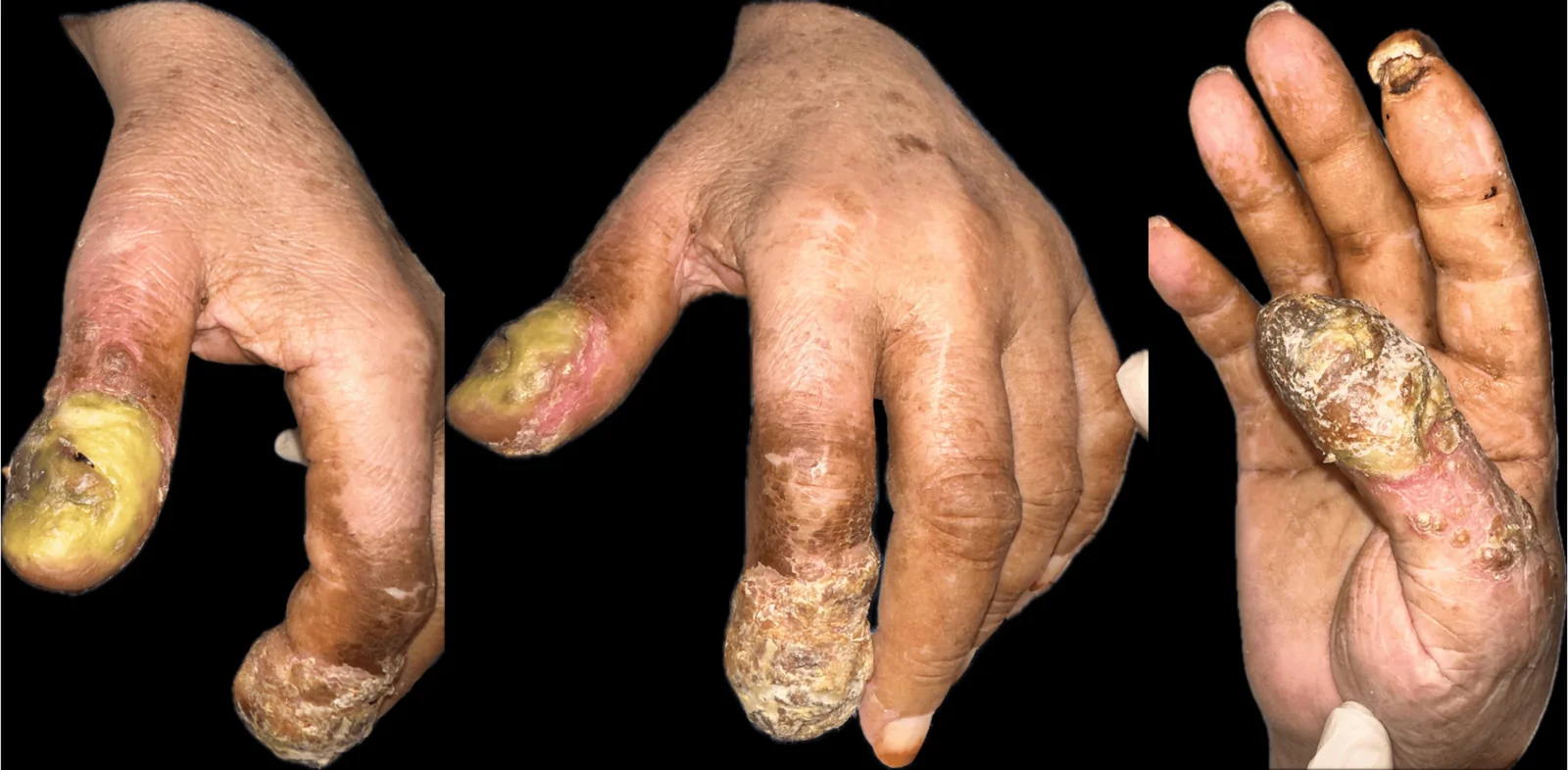
Clinical presentation of severe acrodermatitis continua of Hallopeau Clinical images showing bilateral paronychia with thick yellow-green hyperkeratosis and erythematous, crusted plaques extending proximally along the affected digits toward their bases. Erosive areas are visible at the periphery of the hyperkeratotic lesions.

Contact dermatitis was considered unlikely in the absence of relevant exposure to water, detergents, other known irritants, or potential contact allergens. Chronic fungal paronychia with secondary bacterial superinfection was initially suspected. Laboratory investigations revealed mildly elevated C-reactive protein levels (25 mg/L), with a normal leukocyte count. Bacterial and fungal cultures from purulent material and erosive bed swabs were negative; however, cultures from the extracted hyperkeratotic material grew* Pseudomonas aeruginosa*. Radiography and ultrasonography excluded underlying bone or deep soft tissue involvement.

Initial management included local care with diluted vinegar soaks and thick white petrolatum applied one hour before debridement, followed by topical silver sulfadiazine and light-emitting diode (LED) therapy. This resulted in marked clinical improvement, with clearance of the overlying hyperkeratosis within six days, revealing underlying anonychia and a markedly inflamed, erosive periungual surface bordered by thick whitish desquamation (Figure 2).

**Figure 2:**
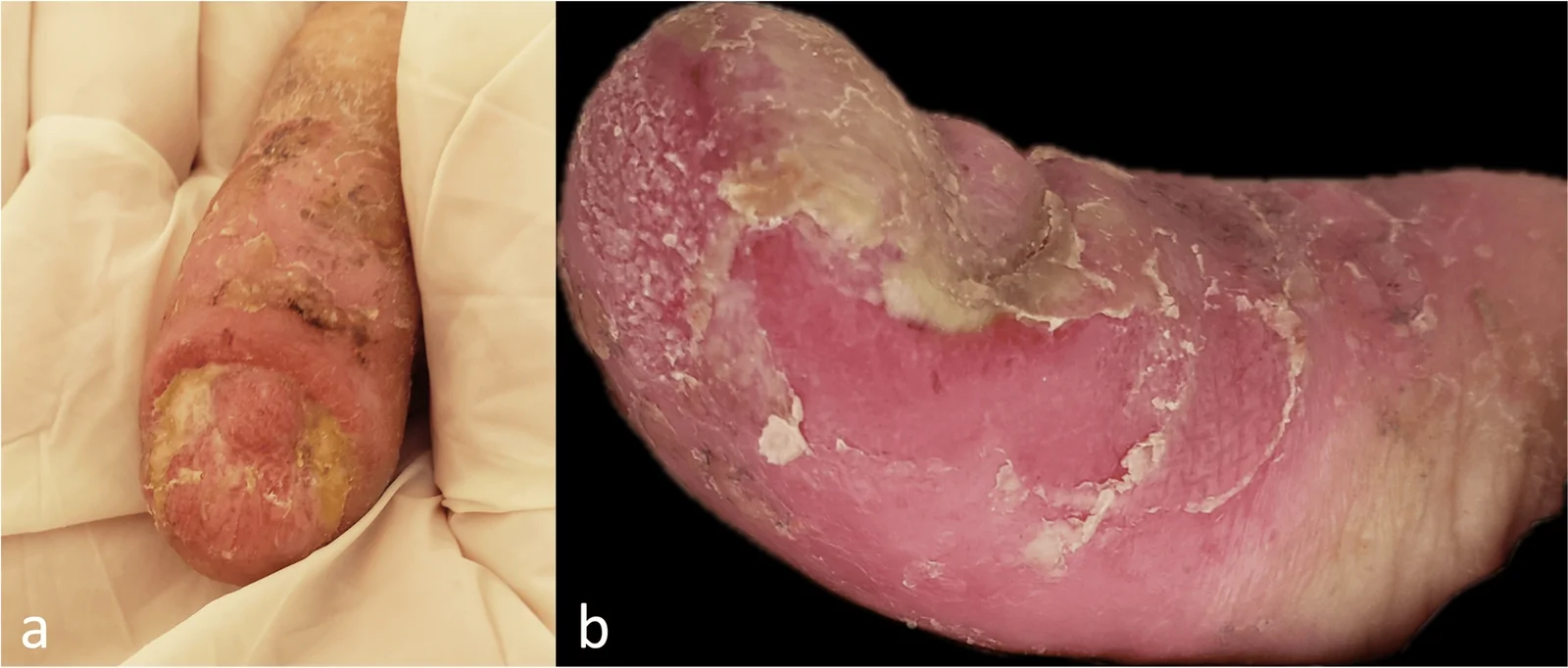
Post-debridement clinical findings (a) Anonychia. (b) Markedly inflamed erosive periungual surface bordered by thick whitish scales.

Periungual dermoscopy demonstrated an erythematous background, large structureless white-yellowish areas corresponding to giant pustules, multiple coalescent milky pustules, yellowish crusts, whitish scales, and shiny reticular white structures enclosing regularly distributed dotted, globular, and glomerular vessels. Small polymorphous white-yellow structures were also noted (Figure 3).

**Figure 3:**
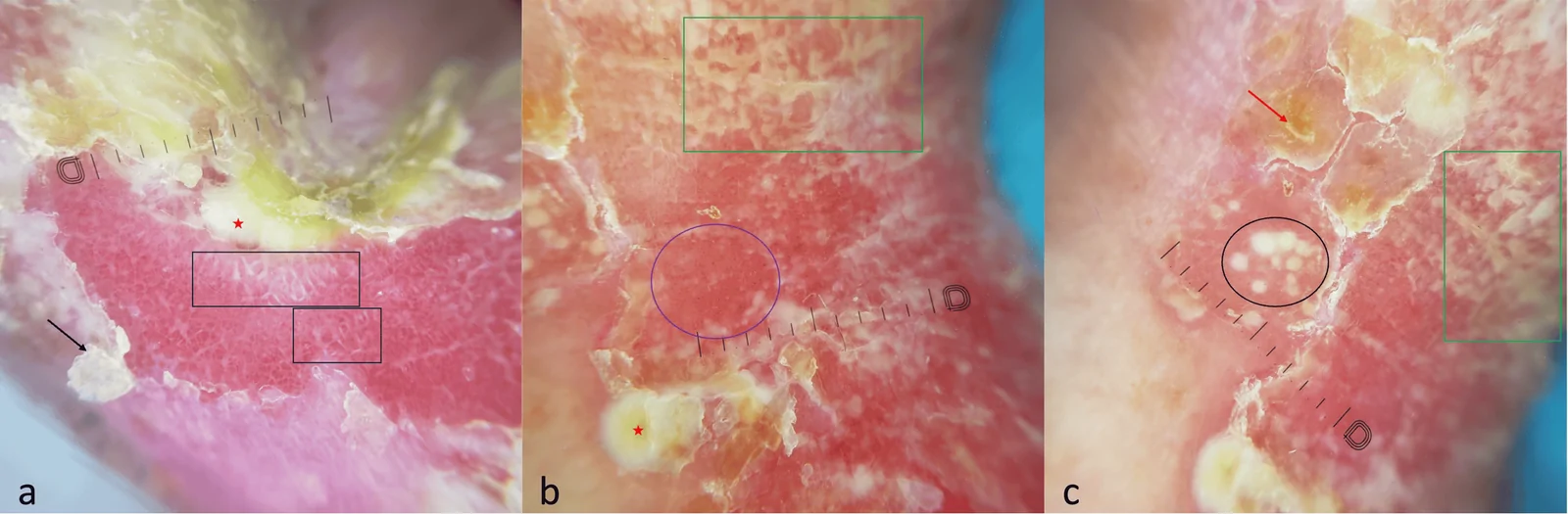
Polarized contact dermoscopy of the periungual folds (DermLite DL5, ×10) Dermoscopic images showing an erythematous background with giant pustules (red stars, a, b), coalescent milky pustules (black circle, c), yellowish crusts (red arrow, c), whitish scales (black arrow, a), and shiny reticular white structures (black squares, a), associated with regularly distributed dotted, globular, and glomerular vessels (violet circle, b) and small polymorphous white-yellow structures (green squares, b, c).

Based on the clinicodermoscopic findings, a periungual skin biopsy was performed to differentiate ACH from nail erosive lichen planus (NELP). Histopathology revealed laminated parakeratosis, psoriasiform acanthosis with elongated rete ridges, and Munro microabscesses. The papillary dermis showed dilated capillaries, mild edema, and a mixed lymphohistiocytic and neutrophilic infiltrate. No dermoepidermal interface changes were identified (Figure 4). A final diagnosis of ACH with secondary bacterial superinfection was established.

**Figure 4:**
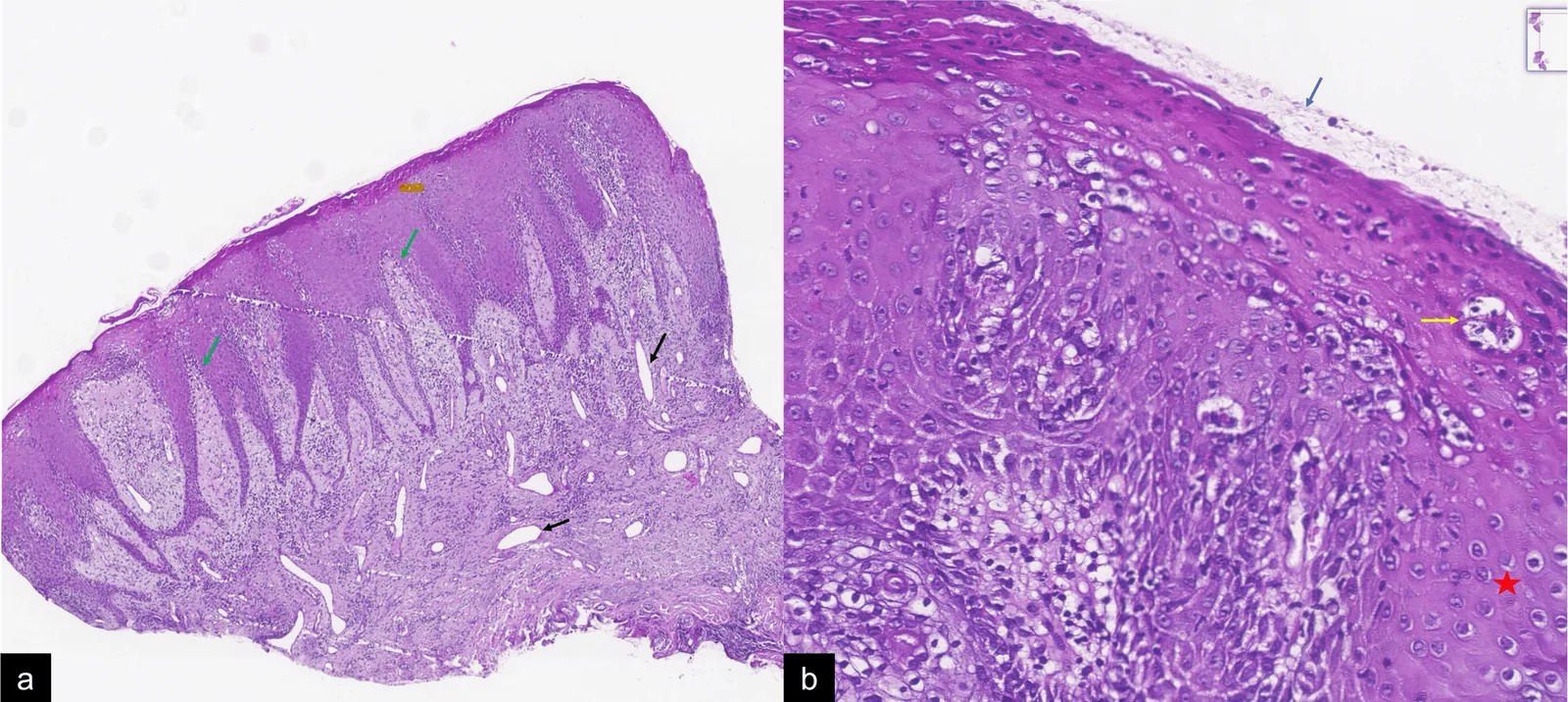
Histopathological features of a periungual biopsy (a) H&E ×40 demonstrating regular psoriasiform acanthosis with elongation of rete ridges (green arrows) along with dilated capillaries in the papillary dermis (black arrows). (b) H&E ×400 revealing parakeratosis (blue arrow), Munro microabscesses (yellow arrow), and a mixed lymphohistiocytic and neutrophilic inflammatory infiltrate (red star).

The patient was started on twice-daily topical betamethasone/calcipotriol combination gel, pending pre-acitretin evaluation. Unfortunately, he subsequently sustained a femoral neck fracture complicated by cardiac decompensation and died shortly thereafter.

## Discussion

ACH is a rare, chronic, and relapsing form of localized PP. It predominantly affects adults, with a female preponderance and a mean age of onset around 40 years [2,4]. Typically, one to three digits are involved, most commonly the thumb, with fingers more frequently affected than toes [2]. Multiple digits and, rarely, all 20 nails may be involved [4].

ACH is characterized by infiltration of neutrophils into the epidermis, forming sterile pustules, similar to GPP and PPP. These three variants differ phenotypically from each other and from psoriasis vulgaris (PV), although they may coexist [4]. According to ERASPEN, ACH is classified as a distinct entity separate from GPP, with the nail apparatus considered the key structure for diagnosis. Mixed forms are classified according to the predominant clinical entity, while pustules outside the nail apparatus are considered PPP or undifferentiated pustules [1,5]. In contrast, Japanese guidelines consider ACH a GPP subtype [4]. Like other PP variants, ACH belongs to autoinflammatory keratinization diseases (AIKDs), with genetically driven innate immune hyperactivation causing epidermal and dermal inflammation and hyperkeratosis [4].

Clinically, ACH begins with erythema at a digit tip, followed by tender pustule formation in periungual/subungual regions. Pustules are irregular in shape with ill-defined borders and may coalesce into "lakes of pus". Initially, the nail plate may remain intact, allowing subungual pustules to be visible, but over time it often thickens and becomes opaque, deformed, and covered with yellow-brown crusts [4]. Chronic disease may progress proximally to the dorsal hand or foot and lead to progressive nail dystrophy, atrophy, and eventual anonychia, leaving a shiny, erythematous, smooth distal digit where pustules frequently recur [2,5]. Affected digits may develop hyperkeratosis with psoriasiform scaling. Rarely, osteitis can cause osteolysis of the underlying phalanges in severe, long-standing cases. Very rarely, acquired syndactyly has been reported, likely due to loss of interdigital epidermis allowing fusion during healing [5]. Squamous cell carcinoma (SCC) has also been reported on chronically inflamed ACH lesions [4]. ACH can even evolve into GPP in approximately 13% of cases, particularly the life-threatening von Zumbusch type, after months or years of nail involvement (mean: 2.2±1.7 years) [1].

Minor trauma, local infection, and neurological and inflammatory etiologies have been reported as triggering factors. Unlike GPP, corticosteroid withdrawal, menstruation, and pregnancy are not major triggers. In quiescent stages, pustules may be absent, making history of recurrent painful flares an important diagnostic clue [1,2,4].

Comorbidities in ACH are similar to those seen in GPP, with metabolic disorders commonly observed [1]. Concurrent PV occurs in approximately 20-30% of cases, while psoriatic arthritis (PsA) is present in 20-40% of patients, the highest prevalence among PP variants [4].

ACH is frequently misdiagnosed as bacterial, fungal, or viral paronychia. Other differential diagnoses include secondarily infected contact dermatitis, dyshidrotic eczema, autoimmune bullous disorders (e.g., pemphigus vulgaris or vegetans), paraneoplastic syndromes, and SCC in monodactylic cases [2].

As biopsy of the distal digits is often technically challenging and uncomfortable for the patient, dermoscopy represents a valuable non-invasive diagnostic tool in such cases. Typical dermoscopic findings in ACH include whitish-yellow hyperkeratosis or scaling, pustules, dotted or linear vessels, and hemorrhagic spots [3,6]. Regularly distributed dotted vessels may also be observed [3].

However, atypical dermoscopic features, as seen in our case, may mimic NELP or candidiasis, thereby creating diagnostic pitfalls. NELP is a rare variant of erosive lichen planus characterized by nail bed erosions, acute paronychia, and permanent anonychia [7]. In our patient, reticular shiny white structures were observed. However, the absence of hypergranulosis on histopathologic examination ruled out true Wickham striae, and these structures most likely corresponded to underlying acanthosis. Additionally, small round yellow-white areas on an erythematous background are considered relatively specific for candidiasis [8]. However, potassium hydroxide examination was negative in our patient, suggesting that these dermoscopic features were more likely related to ruptured pustules. Secondary infection with* Pseudomonas aeruginosa *and underlying onychomycosis may have acted as precipitating factors for the flare in this case.

Histopathologically, ACH resembles PP, with subcorneal and spongiform pustules composed predominantly of neutrophils, accompanied by parakeratosis and a moderate lymphohistiocytic infiltrate. Mast cells and occasional eosinophils may be observed around the acrosyringium. In chronic stages, thinning of the epidermis and atrophy of the papillary dermis may occur. The nail matrix is consistently involved, showing acanthosis, spongiosis, and lymphocytic and neutrophilic exocytosis. Dilated and tortuous capillaries are frequently present within the nail bed and matrix [2,4,5].

Pustule cultures are generally sterile, although commensal organisms may occasionally be isolated without pathogenic significance [5]. Overall, ACH is considered a neutrophilic dermatosis, in which persistent spongiform pustulation can lead to nail bed and matrix destruction, resulting in permanent atrophy.

Plain radiographs help assess osteolysis, while ultrasound may identify associated PsA. Erosive changes typically involve the distal phalanx without affecting the distal interphalangeal joint, distinguishing ACH from PsA [4].

Recent studies suggest genetic contributions to ACH. Mutations in IL-36RN (encoding IL-36Ra) impair the inhibition of IL-36 signaling, leading to neutrophil aggregation and pustule formation. Loss-of-function mutations upregulate IL-36, IL-1, IL-8, IL-17, and TNF-α pathways. TNF-α amplifies IL-36 effects, while IL-17 modulates downstream inflammation. These mutations are found in ~20% of ACH cases, mostly biallelic, and tend to be hypomorphic compared to GPP, explaining the more localized phenotype. IL-36RN mutations are associated with earlier onset, more severe phenotype, and higher systemic inflammation risk. Other implicated genes include CARD14 and AP1S3 [1,4].

ACH is chronic and refractory, with high rates of relapse. Spontaneous improvement is uncommon [2,4]. Systemic therapy is recommended even for single-digit involvement, while topical agents are primarily adjunctive or for maintenance. First-line systemic therapy includes acitretin (0.5 mg/kg/day), with reported efficacy in ~30% of cases. Methotrexate (15 mg/week) is less effective (~20%), while cyclosporine (3-5 mg/kg/day) is useful for short-term control in severe cases. Topical options include corticosteroids, alone or in combination with calcipotriol, calcipotriol alone, and tacrolimus. Corticosteroids should be limited (<21 days) to prevent skin and bone atrophy, while tacrolimus and calcipotriol may be used long-term but discontinued if ineffective after six months. Intralesional triamcinolone (5-10 mg/mL, 0.1-0.2 mL per nail quadrant) is an alternative but may be painful [4].

Second-line therapy involves biologics, guided by genetic insights. IL-36RN mutations predict more severe disease, and IL-36-driven inflammation may respond to IL-36 inhibitors like spesolimab. IL-17 and IL-23 inhibitors are useful in the presence of CARD14 or AP1S3 mutations. TNF-α inhibitors also showed good responses. Treatments targeting IL-12 or IL-23 and IL-1 signaling seem to be less effective. Response rates vary, and biologics are only slightly superior to conventional therapies. Small molecules (apremilast, JAK, and TYK2 inhibitors) show variable short-term efficacy but limited long-term data [1,2,4,9,10].

Pain is a hallmark of ACH, often unresponsive to over-the-counter anti-inflammatories. Nimesulide (200 mg/day) has been effective in acute phases, though prolonged use raises liver toxicity concerns. Low-dose oral naltrexone (3-5 mg/day) may offer a safer alternative [4].

## Conclusions

ACH is a rare, chronic, and diagnostically challenging form of localized PP. Atypical clinical presentations, such as massive hyperkeratosis, and misleading dermoscopic features, like reticular shiny white structures mimicking Wickham striae, as observed in our case, can complicate early recognition and delay management. Accurate diagnosis requires careful integration of clinical history, dermoscopy, histopathology, and microbiologic studies. Prompt identification and individualized systemic therapy are essential to prevent progressive nail destruction, bone involvement, functional impairment, and potential systemic complications. Clinicians should remain vigilant for unusual presentations, particularly in elderly patients with chronic, destructive paronychia.
